# Mahanine drives pancreatic adenocarcinoma cells into endoplasmic reticular stress-mediated apoptosis through modulating sialylation process and Ca^2+^-signaling

**DOI:** 10.1038/s41598-018-22143-w

**Published:** 2018-03-02

**Authors:** Sayantani Sarkar Bhattacharya, Chandan Mandal, Reinhard Schwartz Albiez, Suman Kumar Samanta, Chitra Mandal

**Affiliations:** 1 Cancer Biology and Inflammatory Disorder Division, Council of Scientific and Industrial Research-Indian Institute of Chemical Biology, Kolkata, 700032 India; 2 German Cancer Research Center Heidelberg, Antigen Presentation & T/NK Cell Activation, Clinical Cooperation Unit Applied Tumor Immunity, ImNeuenheimer Feld 280, D-69120 Heidelberg, Germany

## Abstract

Endoplasmic reticulum (ER) stress results from protein unfolding/misfolding during cellular maturation, which requires a coordinated action of several chaperones and enzymes and Ca^2+^ signalling. ER-stress possibly has a positive effect on survival of pancreatic cancer cell. Therefore, detailed insights into this complex signaling network are urgently needed. Here, we systematically analyzed the impact of ER stress-mediated unfolded protein response (UPR) and Ca^2+^-signaling cross-talk for the survival of pancreatic adenocarcinoma (PDAC) cells. We observed enhanced ER activity and initiation of UPR signaling induced by a carbazole alkaloid (mahanine). This event triggers a time-dependent increase of intracellular Ca^2+^ leakage from ER and subsequently Ca^2+^ signaling induced by enhanced reactive oxygen species (ROS) produced by this pro-oxidant agent. In addition, we observed an altered glycosylation, in particular with regard to reduced linkage-specific sialic acids possibly due to decreased sialyltransferase activity. Changes in sialylation entailed enhanced expression of the ganglioside GD3 in the treated cells. GD3, an inducer of apoptosis, inhibited pancreatic xenograft tumor. Taken together, our study describes a molecular scenario how PDAC cells are driven into apoptosis by mahanine by UPR-driven ER stress-associated and ROS-mediated calcium signaling and possibly defective sialylation.

## Introduction

Initial protein maturation steps take place in the endoplasmic reticulum (ER), which involves folding, assembly, quality control of secretory and membrane proteins, disulfide bond formation, initial steps of glycosylation and lipid biosynthesis^1^. In addition, ER is the major intracellular organelle for calcium storage^2^. Under stress conditions, when the protein-folding ability is inundated, unfolded or misfolded proteins are accumulating in the lumen which leads to ER stress^3^. To relieve stress and re-establish the cellular homeostasis, the ER activates an array of intracellular signal transduction pathways, collectively termed as unfolded protein response (UPR) which is critical for the maintenance of cellular function. This UPR reduces the influx of newly synthesized proteins into the ER through general translational arrest, induces the transcriptional upregulation of genes, in particular, those of distinct chaperones which enhance protein folding capacity and quality control. Also, UPR induces degradation of proteins with aberrant conformation through the proteasome (ER-associated degradation, ERAD) and lysosome-mediated autophagy^4–6^.

Pancreatic ductal adenocarcinoma (PDAC) is the twelfth most common type of cancer and seventh most common cause of death in the world^7^. The 5-year survival rate is only 7.7%^8^. Due to an increased occurrence and poor prognosis and inadequate opportunity to improve overall survival, PDAC is anticipated to be the second-leading cause of cancer-related death by 2030^9^.

Due to the inadequate availability of a functional vascular supply, the tumor micromilieu of pancreatic tumors is deficient in important metabolites^10^. This tumor micro-environment provides conditions for predisposing tumors to ER stress. Several studies have connected protein kinase RNA-like ER kinase (PERK) signaling with enhanced tumor growth and survival under hypoxic environment^11^. Molecular evidence of PERK activation in human primary cancers including melanomas, glioblastomas, breast and cervical cancers are reported. In addition, ER stress-mediated apoptosis, including proteasomal inhibitors and cisplatin as inducing agents, has been reported^12,13^. Thus, new therapeutics targeting PERK to inhibit its influence on UPR are under investigation^11–15^. Up to now, it is unclear how tumor cells balance the beneficial versus cytotoxic outputs derived from PERK signaling. Thus, there may be multiple diverse mechanisms by which ER stress may favor malignant transformation.

Therefore, ER stress-mediated UPR plays a dual role both in apoptosis and survival in cancer. As a result, one problem with the UPR targeting agents is perhaps the difficulty to identify a critical therapeutic index between the cytoprotective versus apoptotic effects of ER-stress induction. ER stress-stimulating agents may be exploited to enhance threshold level of basal ER stress as much like the pro-oxidant agents act in cancer cells. Hence, they possibly prove to be a new modality for cancer treatment.

Sialic acids are mainly terminal *N*- and *O*-substituted 9-carbon monosaccharides and play various cellular functions^16–22^. Malignancy with metastatic properties is directly linked with altered sialylation pattern^23–28^. Amongst several enzymes responsible for sialylation of glycoprotein, sialyltransferase transfers CMP-activated sialic acid to the terminal glycoprotein while sialidases, other key enzymes of sialic acid catabolism cleave the terminal sialic acid from sialoglycoprotein. Therefore, balance between these two key enzymes plays a critical role in the fate of a sialoglycoprotein^29–31^.

Gangliosides play important roles in the development, differentiation, and proliferation of mammalian cells. They bind to other cell membrane components through their terminal sialic acids. GD3, an inducer of apoptosis, is often represented as a tumor-related ganglioside in many cancers including glioma, colorectal carcinoma, sarcoma, and leukemia. Latest works from our laboratory reported GD3-induced apoptosis in PDAC^32^.

We have recently demonstrated that a pro-oxidant carbazole alkaloid, mahanine, isolated from a dietary plant *Murraya koenigii*, could impair the functional activity of heat shock protein 90 (Hsp90) in PDAC in reactive oxygen species (ROS)-dependent manner^33^. Accordingly, we hypothesized that this accumulated ROS could also increase the protein load within the cell predominantly within the ER that may ultimately initiate UPR. We have observed that this pro-oxidant agent not only turned on ER stress signaling by modulating its molecular mediators but also altered Ca^2+^ pool and signaling in the PDAC cell. Additionally, it changed glycosylation profile by modulating key enzyme in this treated cell.

## Results

### Escalation in ER activity in PDAC cells

**Figure 1:**
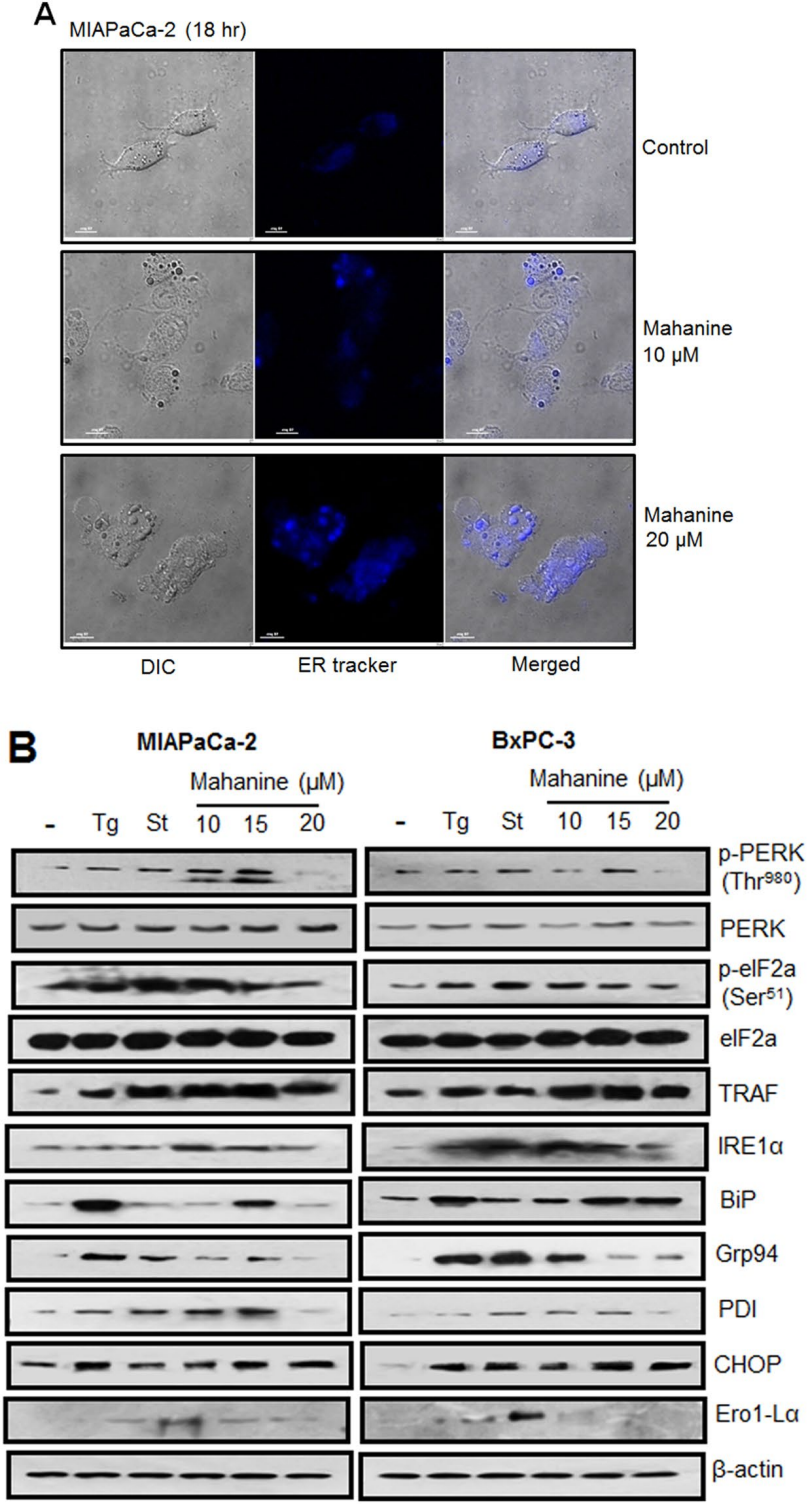
Escalation in ER activityin mahanine-treated MIAPaCa-2 cell. (**A**) ER activity was visualized by confocal microscopy stained with ER-tracker blue in MIAPaCa-2 cell treated with the different dose of mahanine for 18 hr. (**B**) Enhanced unfolded protein response (UPR) related proteins in the cell lysate of two representative pancreatic carcinoma cells (MIAPaCa-2 and BxPC-3) treated with thapsigargin (Tg, 1.0 µM), staurosporine (St, 1.0 µM) and with increasing doses (10, 15 and 20 µM) of mahanine for 18 hr by western blot analysis.

As earlier described, we observed that mahanine induces ROS leading to the Hsp90 dysfunction in PDAC cells^33^. Hence, we hypothesized that this event may raise the protein load within the cell, predominantly in the ER. Therefore, we analyzed whether accumulated ROS increases stress in the ER compartment. To study the activity of ER, MIAPaCa-2 cells were incubated with prooxidant mahanine (10 µM and 20 µM) for 18 hr and stained the cells with ER-Tracker Blue-White DPX dye (Fig. 1A). This photostable dye is selective for the ER in live cells and yields blue staining. Treated cells exhibited significantly increased blue staining compared to the control suggesting the accelerated activity of ER and ultimately induced ER stress. This result confirmed that mahanine-induced ROS can induce ER activity in PDAC cells *in vitro*.

### Enhanced transcripts involved in classical unfolded protein response pathway

Next, we studied whether mahanine could also augment UPR through ROS in PDAC cells. The microarray analysis demonstrated that most of the classic UPR transcripts were increased (Fig. S1, Table S1). The transcripts involved in canonical UPR pathway were increased significantly (p < 0.005,one-way ANOVA was used) in treated cells. Among these, activating transcription factor 4 (ATF4) transcript 1 and tumor necrosis factor (TRAF7) were upregulated maximally at about 1.8 fold and 1.6 fold respectively at 18 hr. SERCA2 and XBP1 transcripts increased more than 1.4 fold as well as ATF4 transcript 2, PDIA6, ERdj5and TRAF4 increased more than 1.2 fold at this time point. In addition, a few known ER-stress responsive genes namely CHOP, Grp94, BiP, and TRAF2 were also upregulated.

### Augmentation of UPR signaling

Next, we explored the status of UPR proteins involved in this signaling cascade in two representative PDAC cells, MIAPaCa-2 and BxPC-3 (Fig. 1B). The results clearly demonstrated upregulation of the PERK phosphorylation at amino acid T^980^ and consequently phosphorylation of eIF2α at the amino acid S^51^ site, confirming that translational initiation process was attenuated. Another UPR protein, IRE1α, was also upregulated in treated cells. This signaling further promoted the upregulation of ER chaperones and protein folding enzyme machinery viz. BiP, Grp94, CHOP, and ERO1Lα which proved that mahanine could also activate this pathway. In parallel, upregulation of TRAF2 and consequent cleavage of caspase 12 additionally proved the activation of second downstream signaling of IRE1. Thus phosphorylation of PERK eventually led to the phosphorylation of the eukaryotic translation initiation factor eIF2α and finally attenuated the translation process in both the cells.

### Alteration in gene expression of Hsp40 homolog family in MIAPaCa-2 cells

DnaJ/Hsp40 family proteins have been preserved throughout evolution and are important for protein translation, folding, unfolding, translocation and degradation. They primarily stimulate the ATPase activity of Hsp70s to protect client proteins from irreversible aggregation during synthesis and in times of cellular stress^34^. While studying the status of the DnaJ family transcripts from the microarray data, we observed upregulation of most of the proteins of this family (Fig. S2a). Hsp40 homolog proteins DnaJC5G was significantly upregulated (about 1.8 fold), already after 18 hr of incubation with mahanine (Fig. S2b). These data also reconfirmed that mahanine-induced ROS led to cellular stress.

**Figure 2:**
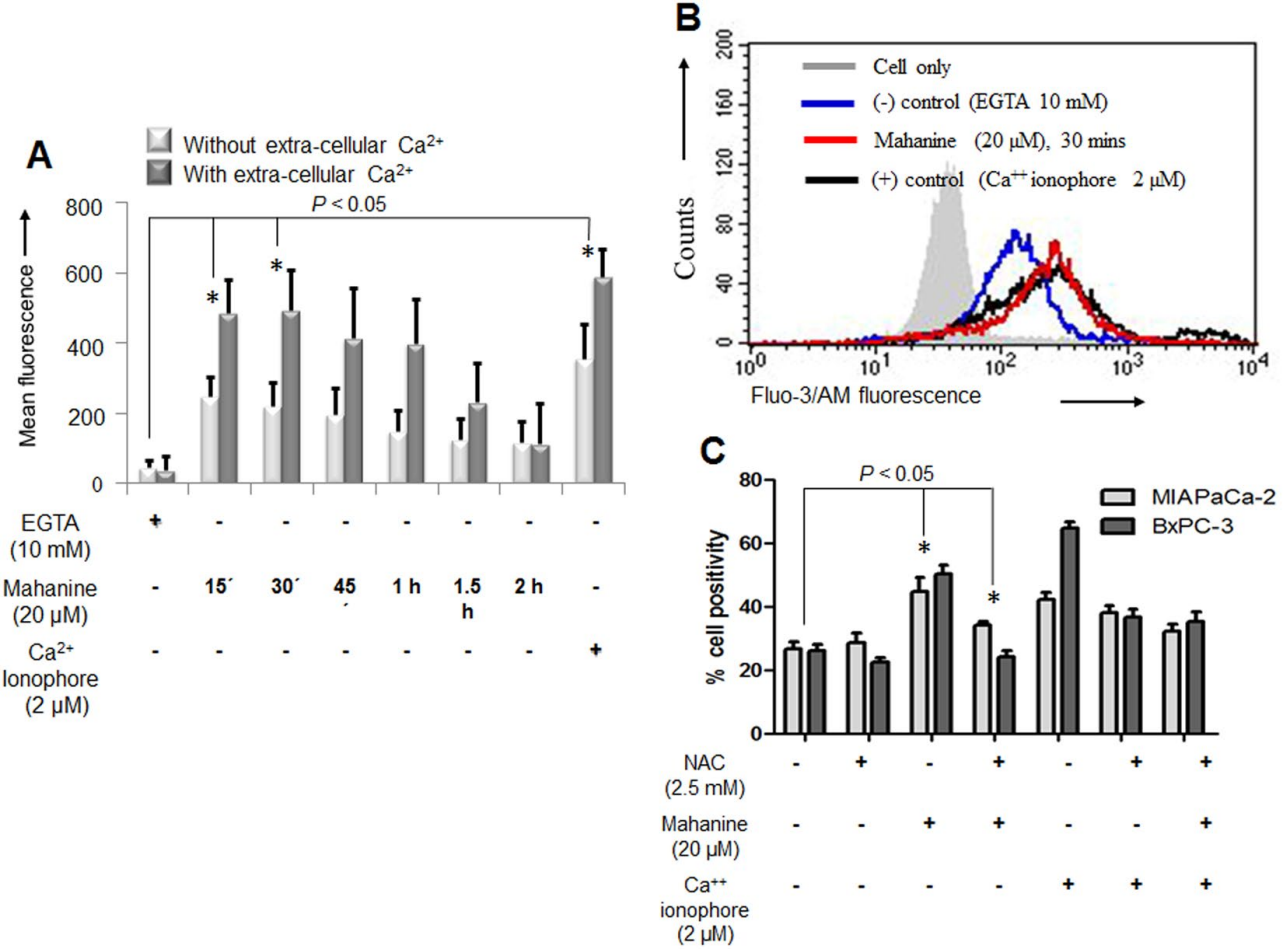
Mahanine-treated MIAPaCa2 cell exhibited increased intracellular Ca^2+^. (**A**) Histogram plot of the mean fluorescence intensity of intracellular Ca^2+^ pool as measured by Fluo-3/AM in MIAPaCa-2 cells treated with mahanine (20 µM) at different time points. (**B**) A representative Fluo-3/AM MFI (mean fluoresce intensity) spectra in mahanine-treated MIAPaCa-2 cells. EGTA (10 mM) and Ca^2+^ ionophore (2 µM) were served as the negative and positive controls respectively. (**C**) Measurement of Ca^2+^ leakage mediated by ROS in MIAPaCa-2 and BxPC-3 cells pretreated with NAC (ROS inhibitor, 1 hr) followed by mahanine and Ca^2+^ ionophore with indicated dose for 30 min by FACS.

### Time-dependent increase of intracellular Ca^2+^ in MIAPaCa-2 cells

Inhibition of chaperones can lead to the augmentation of protein load as part of the ER stress process. This ER stress can also lead to the leaching out intra-cellular Ca^2+^ from ER. Accordingly, we next wanted to evaluate the intracellular Ca^2+^ pool in treated cells (Fig. 2A,B). The maximum enhancement of the intracellular Ca^2+^ pool in MIAPaCa-2 cells was observed within 15 min after treatment, even without the presence of extracellular Ca^2+^. EGTA (10 mM) and Ca^2+^ ionophore (2 µM) served as negative and positive controls where it showed the lowest and the highest mean fluorescence intensity (MFI) respectively. These results confirmed that this prooxidant agent may induce the release of Ca^2+^ from the ER and activate ER stress in these cells. The graphs presented as mean of at least three independent experiments. Each value represented as mean ± SD.

### Ca^2+^ leakage from ER triggered by ROS

Subsequently, we anticipated whether mahanine-induced ROS plays a critical role to activate the Ca^2+^ leakage. Flow cytometric study demonstrated that mahanine-mediated enhanced Ca^2+^ leakage was decreased when cells were pretreated with ROS scavenger, N acetylcysteine (NAC) (Fig. 2C). The decrease was significant, both in MIAPaCa-2 and BxPC-3 cells. In this study, Ca^2+^ ionophore was used as a positive control as it can directly facilitate the transport of Ca^2+^ across the plasma membrane. The graphs presented as mean of at least three independent experiments. Each value represented as mean ± SD.

### Augmentation of intracellular Ca^2+^ concentration induces Ca^2+^ signaling

As we observed an increase in cellular Ca^2+^ concentration, we next examined the status of downstream signaling which may be affected by altered Ca^2+^ level. We first checked the transcripts, which are known to be involved in Ca^2+^ signaling. Most of them were upregulated and the highest was the calcium transporter type 2 C (ATP2C1, more than 1.8 fold) which directs movement of Ca^2+^ ions within and between cells. In parallel, calcium channel (CACNA1C), the protein complex that forms a transmembrane channel through which calcium ions may pass within or between cells, was also upregulated (1.40 fold). Even calpains [CAPN7 (1.40 fold), CAPN1 (1.25 fold), CAPNS2 (1.20 fold)] were and calcium/calmodulin-dependent protein kinase IG (CAMK1G) were upregulated (Fig. S3). When further concentrating on the genes involved in ER calnexin-calreticulin chaperone cycle, we observed that both of them upregulated around 1.2 fold. With the increase of these chaperone transcripts, the ER degradation enhancer mannoside L1 (EDEM1) and L3 (EDEM3) transcripts were also augmented (>1.2 fold) (Fig. S4).

**Figure 3:**
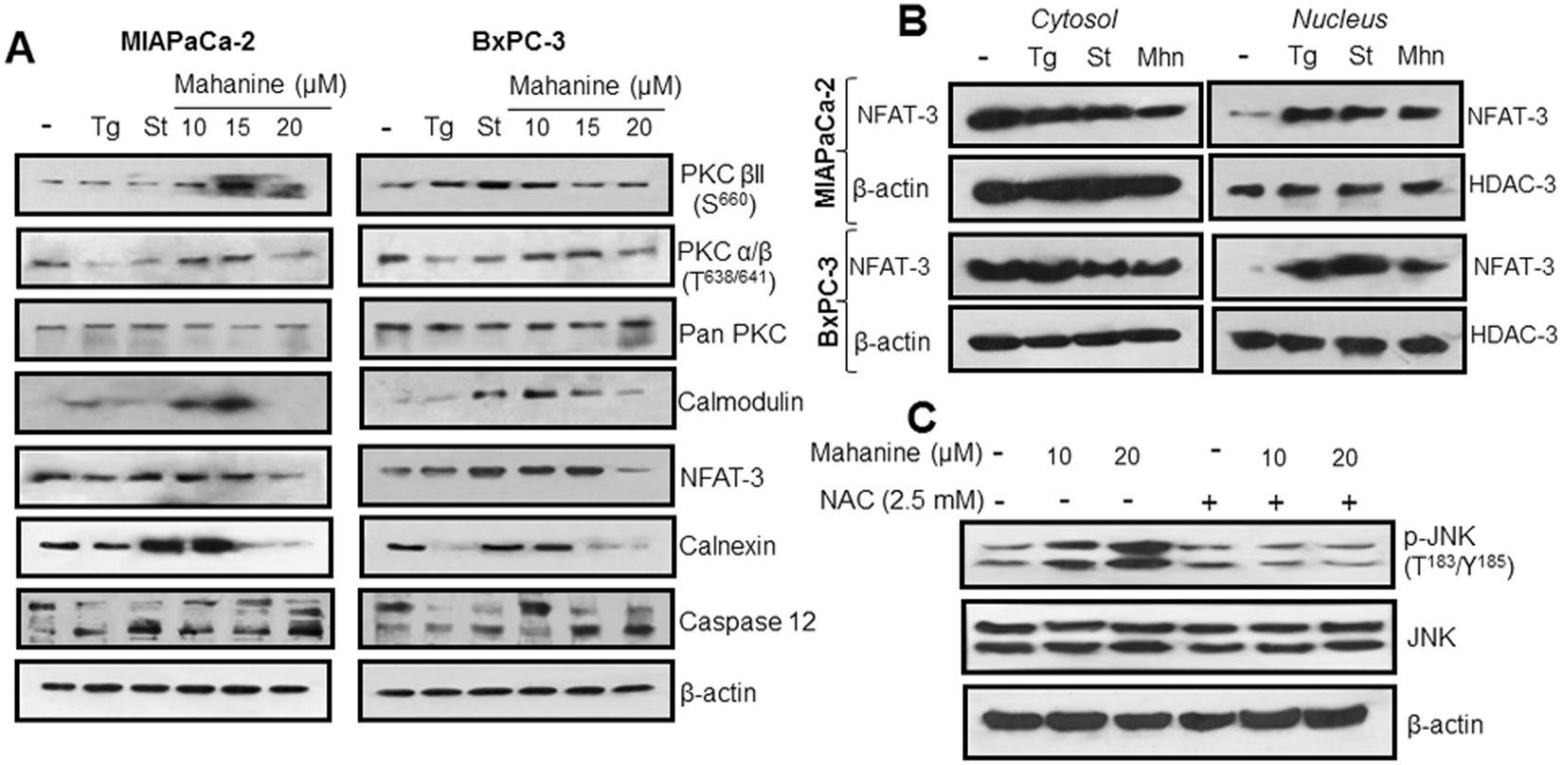
Activation of Ca^2+^ signaling in mahanine treated-pancreatic carcinoma cells. (**A**) Molecular basis of activation of Ca^2+^ signaling was evaluated by western blot analysis in the cell lysate of MIAPaCa-2 and BxPC3 treated with increasing doses of mahanine (10, 15 and 20 µM) for 18 h. (**B**) Translocation of NFAT-3 from cytosol to nucleus in MIAPaCa-2 and BxPC-3 cells treated with 15 µM of mahanine (Mhn) for 18 hr. β-actin and HDAC-3 were used as loading control proteins for cytosolic and nucleus fractions respectively. (**C**) Enhanced phosphorylation of JNK in the MIAPaCa-2 cells treated with increasing doses (10 and 20 µM) of mahanine for 18 hr which was inhibited by pretreatment with NAC for 1 hr indicating the involvement ROS in this process.

In addition to its role in protein synthesis, the ER also orchestrates many signaling events essential for cellular fate, prominent among is Ca^2+^ signaling. Therefore, we subsequently studied different molecular players in this pathway. Likewise, two known ER stress inducers namely thapsigargin (Tg, 1.0 µM) and staurosporine (St, 1.0 µM) were used as controls. PKC βII and PKC α/β were activated in mahanine-treated MIAPaCa-2 and BxPC-3 cells. Calmodulin and its downstream transcription factor NFAT3 were also upregulated. One of the key ER chaperones, calnexin, which ensures the proper folding and quality control of newly synthesized glycoproteins, was also significantly enhanced in these pro-oxidant-treated cells (Fig. 3A). When we further checked whether NFAT-3 could transmigrate from cytosol to nucleus, we observed that prooxidant treatment led to the successful relocation of this transcription factor (Fig. 3B). Activation of ROS mediated JNK, a protein of the MAP-kinase superfamily, was also observed with pro-oxidant treatment (Fig. 3C). These results demonstrate ROS mediated Ca^2+^ leakage from ER which leads the JNK-mediated apoptosis in PDAC.

### Altered glycosylation pattern in PDAC

**Figure 4:**
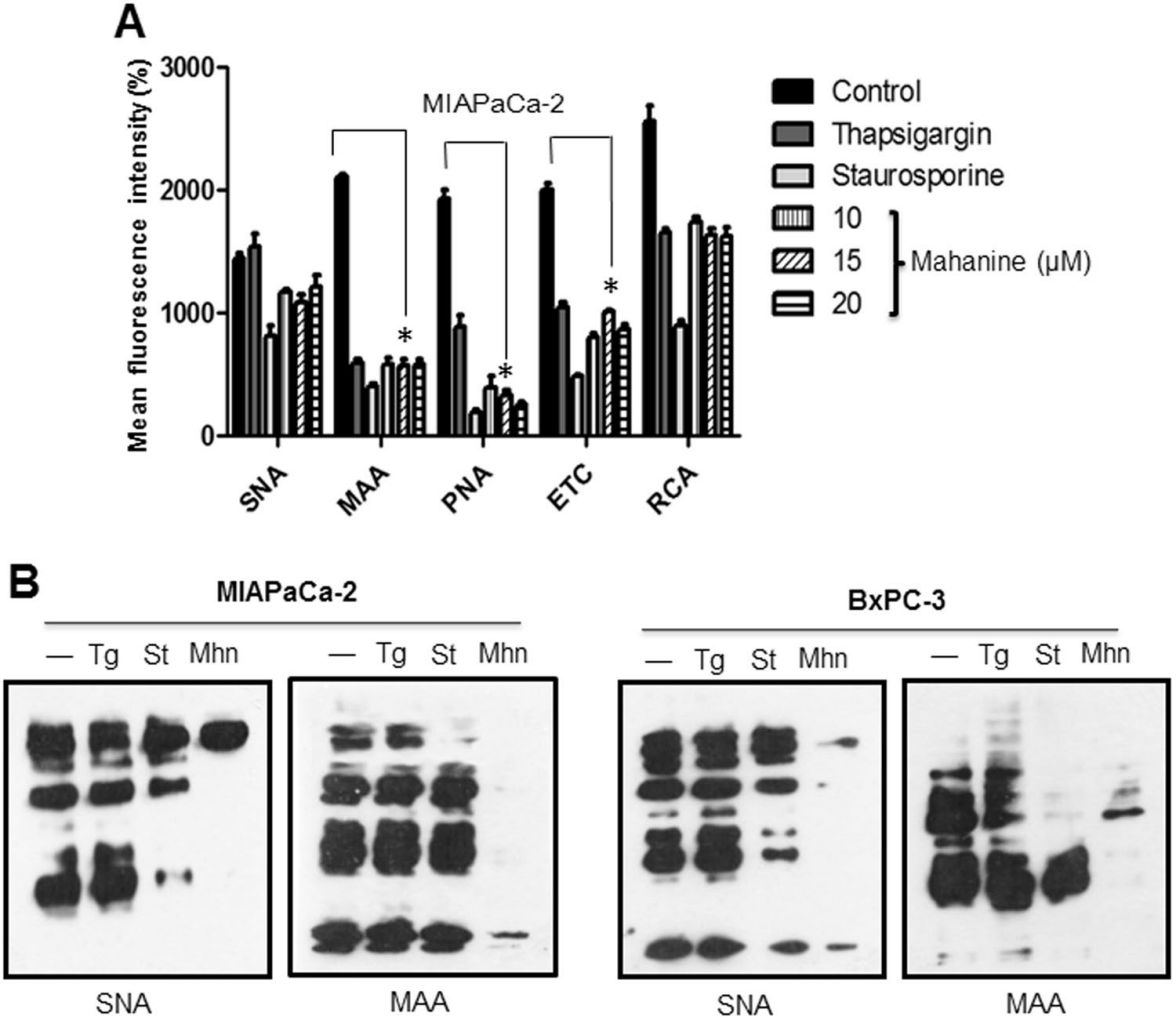
Reduced glycosylation in mahanine treated-MIAPaCa-2 cells. (**A**) Cell surface expression of glycans was determined using different lectins by FACS as described in material and methods. Histogram plot of mean fluorescence intensity (MFI) of different lectins binding towards the MIAPaCa-2 cells treated with different doses of mahanine for 18 hr. (**B**) Evaluation of cellular sialoglycoprotein using two sialic acid binding lectins (SNA and MAA) in the MIAPaCa-2 cells treated with mahanine (Mhn, 20 µM) for 18 hr as described in material and methods. In parallel thapsigargin (Tg, 1.0 µM), staurosporine (St, 1.0 µM) were always used for comparison.

We observed that a prooxidant agent can alter many of the ER function and Ca^2+^ signaling. In addition, we subsequently studied whether the glycosylation process was also changed after mahanine treatment. The results clearly demonstrated that this agent could successfully reduce the expression levels of surface glycoproteins containing terminal α2–6- and α2–3-linked sialic acids, Galβ1–3GalNAc, Galβ1–4GlcNAc and Galβ1–4GalNAc as observed by the decreased binding pattern with different lectins namely SNA, MAA, PNA, ETC and RCA respectively in MIAPaCa-2 (Fig. 4A). Reduction of glycoproteins containing terminal α2–3-linked sialic acids was more prominent. Additionally, thapsigargin and staurosporine-treated cells also exhibited decreased lectin binding. A similar trend was observed in BxPC-3 cells. The graphs presented as mean of at least three independent experiments. Each value represented as mean ± SD.

The sialylation profile of cell surface sialoglycoproteins was further corroborated by western blot analysis using SNA and MAA which recognize terminal α2–6 and α2–3 sialylgalactosyl residues respectively. The result confirmed the drastic reduction in SNA and MAA binding sialoglycoproteins in mahanine-treated MIAPaca-2 cells (Fig. 4B). A similar result was observed in BxPC-3 cells. Two known ER stress inducers, thapsigargin, and staurosporine, also exhibited a similar trend of alteration in SNA and MAA binding, in both cell lines suggesting that the ER stress is responsible for alteration of sialylation.

### Modulation of sialylation controlling enzymatic activity

**Figure 5:**
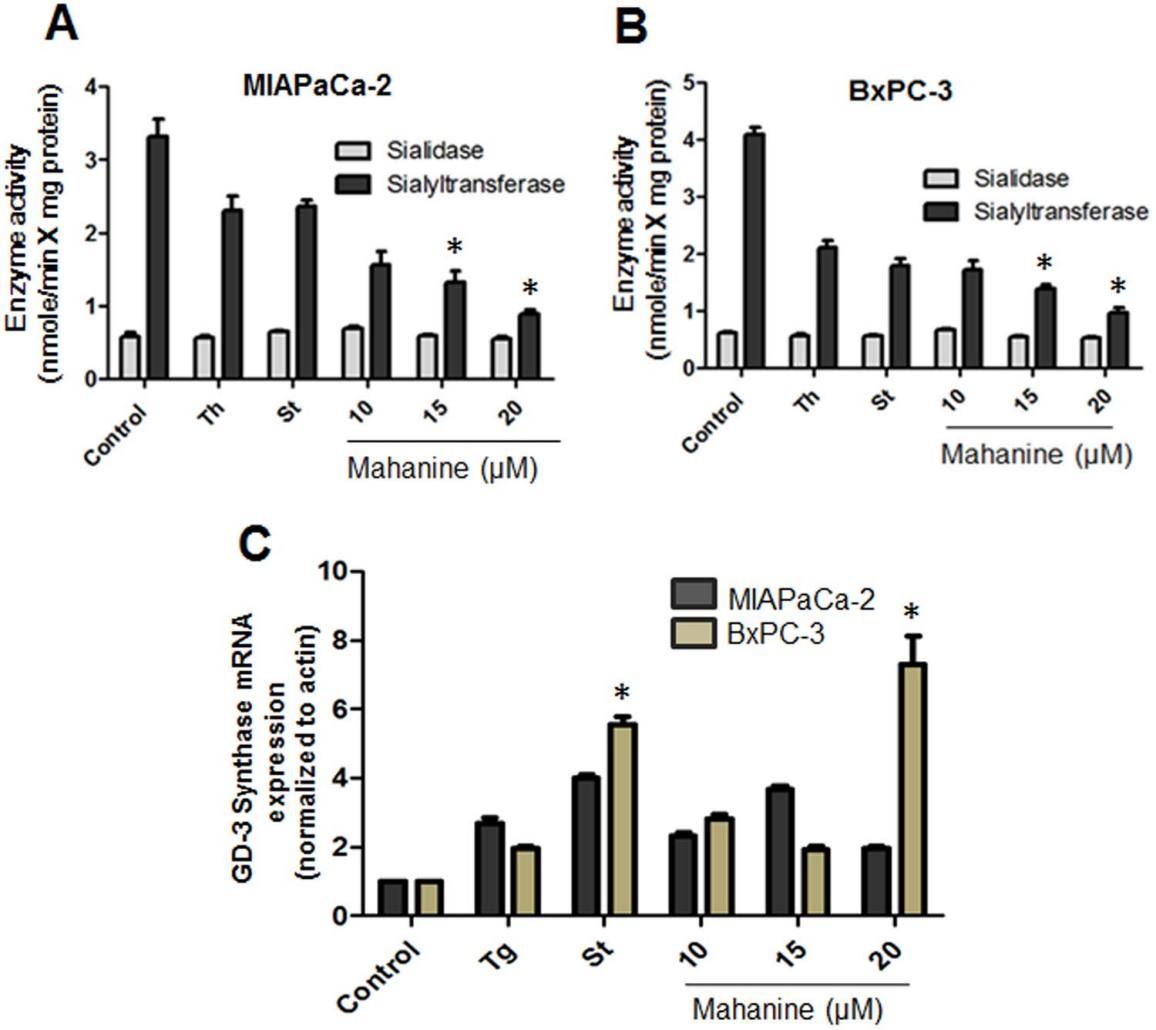
Mahanine modulates sialylation controlling enzymatic activity. (**A**) Enzyme activities (nmol/min X mg protein) of two representative enzymes (sialidase and sialyltransferase) were determined in the MIAPaCa-2 cell treated with mahanine for 18 hr as described in material and methods. (**B**) Sialidase and sialyltransferase enzyme activity (nmol/min × mg protein) in the BxPC-3 cells. (**C**) Relative mRNA level of GD3 synthase gene in MIAPaCa-2 and BxPC-3 cells treated with the increased doses of mahanine for 18 hr.

The decrease in sialoglycoproteins on mahanine-treated cells (Fig. 4A,B) prompted us to further analyze the enzyme activity of sialidase and sialyltransferase (Fig. 5A). The enzyme activity of sialyltransferase was significantly decreased in MIAPaCa-2 cells after mahanine treatment. In contrast, sialidase activity remained unchanged under similar condition. A similar reduction in sialyltransferase activity was also observed in BxPC-3-treated cells (Fig. 5B). Interestingly, both ER-stress inducers (thapsigargin and staurosporine) showed comparatively less effect than mahanine suggesting this prooxidant agent may additionally stimulate some other pathway in MIAPaCa-2 cell. In contrast, these ER stress inducer exhibited similar result like mahanine in BxPC-3 cell. The graphs presented as mean of at least three independent experiments. Each value represented as mean ± SD.

### Mahanine induces the expression of the GD3, the inducer of apoptosis

GD3 is an inducer of apoptosis but often suppressed in cancer. We have recently reported GD3^32^- and mahanine^33^-induced apoptosis in PDAC. Now we intended to study the status of GD3 in the mahanine-treated cells. Despite higher sialylation status (Fig. 5A,B), the disialoganglioside GD3 level is very low both in MIAPaCa-2 and BxPC-3 cells (Fig. 5C). However, there was an enhancement in the level of GD3 transcript both in MIAPaCa-2 and BxPC-3 cells after mahanine incubation. BxPC-3 cells exhibited the highest elevation of GD3. Tg and St also showed similar result suggesting a possible connection between ER-stress and GD3. The graphs presented as mean of at least three independent experiments. Each value represented as mean ± SD.

### Mahanine inhibits pancreatic xenograft tumor

**Figure 6:**
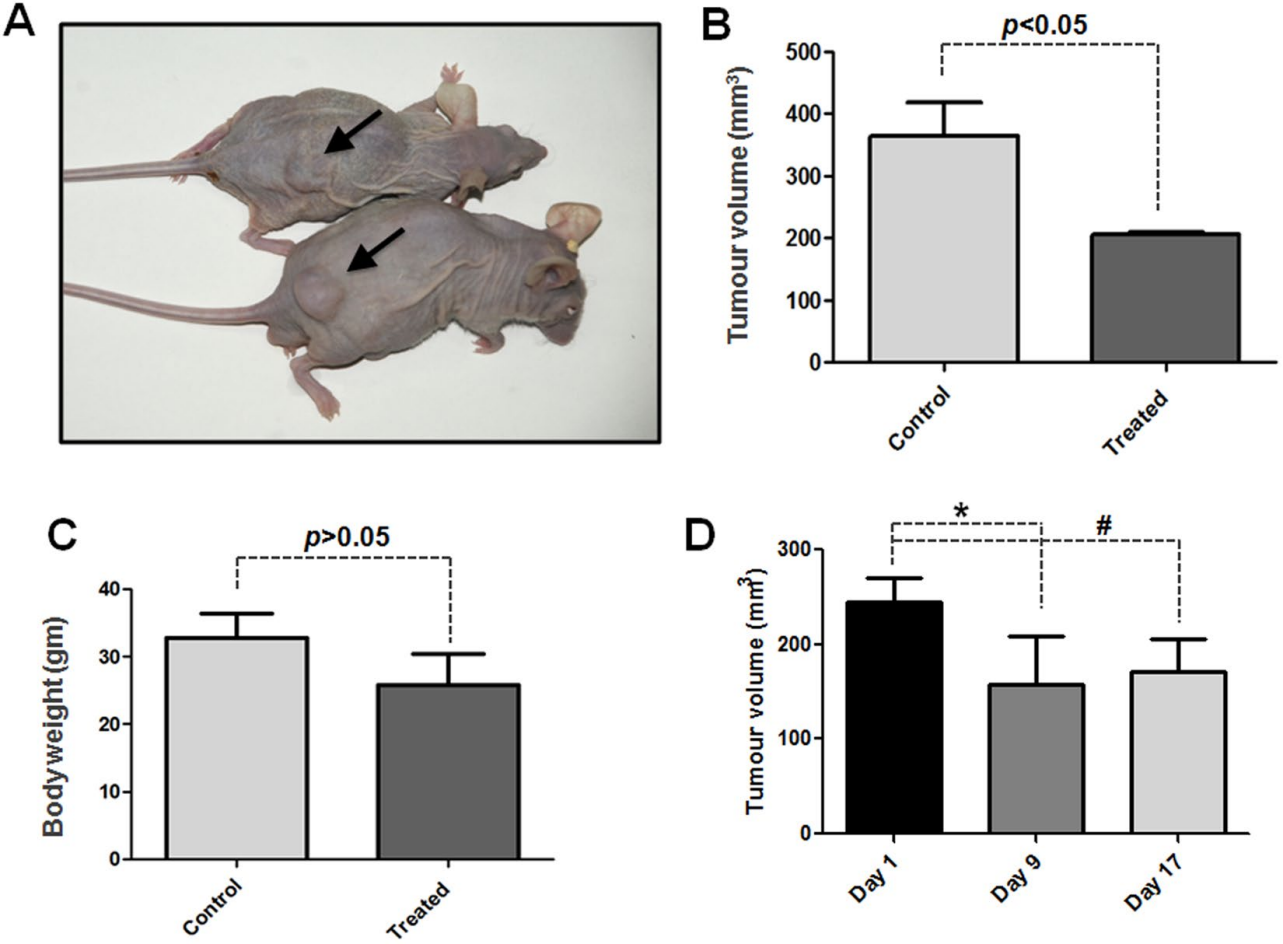
Mahanine inhibits pancreatic xenograft tumor. (**A**) Reduction in tumor volume indicated by arrow (**B**) Significant volumetric reduction (*P < 0.05) measured by the digital vernier calipers with thetreatment of mahanine as described in material and methods. (**C**) No significant difference in total body weight (*P > 0.05) between treated and control group. (**D**) Tumor response graph in different time of treatment (*P < 0.05, ^#^*P* < 0.01).

To further study the activity of mahanine in PDAC *in vivo*, we modeled pancreatic xenograft tumor in sixteen nu/nu mice as described in Materials and Methods section. The results clearly demonstrated that the tumor load is visibly regressed in mahanine-treated nude mice (Fig. 6A). There was a significant reduction (p < 0.05) in tumor volume (Fig. 6B) as measured by the digital Vernier Calipers. However, no significant difference (p > 0.05) in body weight was found in the vehicle control and mahanine-treated mice (Fig. 6C). The tumor response histogram in day 1, day 9 and day 17 of the treatment shows significant reduction in tumor volume. However, the reduction rate decreased in third week of treatment in comparison to the beginning of the treatment (Fig. 6D).

## Discussion

Cancer cells have several survival strategies at their disposal to circumvent assaults by the obnoxious environment. Tumor microenvironments with physiologic ER stress by extreme hypoxia increases tolerance and may even promote tumor growth^10^. Since chaperones of the Hsp family are mainly responsible for protein folding and stability, together with our observation that mahanine-induced Hsp90 dysfunction is due to oxidative insult^33^, we further hypothesized that this carbazole alkaloid also helps to piles up unfolded protein load in the PDAC cells.

We described the activation of UPR-mediated ER stress in two mahanine-treated cell PDAC lines MIAPaCa-2 and BxPC-3. It also affected ROS-induced Ca^2+^ leakage from ER and activation of Ca^2+^-signaling causing altered homeostasis of an intracellular Ca^2+^ pool which potentially further entails ER stress. Moreover, such treatment decreased linkage-specific membrane bound sialic acids along with enhanced GD3 expression. All these events may play an important role in the apoptosis of these cells by a nontoxic carbazole alkaloid isolated from an edible Indian medicinal plant. Taken together, to the best of our knowledge, this is the first report that a prooxidant agent leads to entire ER dysfunction by means of UPR, misbalanced Ca^2+^ pool and altered sialylation in PDAC cells (Figs 4 and 5).

The UPR plays a vital cytoprotective role during ER stress and activates several pathways that ultimately help to attenuate translational machinery, augments chaperone production, and enhances proteasomal degradation^33^. Accumulation of unfolded proteins within the ER induces a set of proteins to facilitate the rate for correct folding. Induction of BiP, CHOP, PDI and other ER stress-related proteins have been widely used as markers of ER stress and the onset of UPR^34^. As live cell imaging of mahanine-treated cells gave a glimpse of escalated ER activity in PDAC cells, we further studied the detail of molecular alterations through microarray analysis. SERCA2, which is established as ER stress-inducible protein^35^, was upregulated significantly after treatment. In parallel, one of like other DnaJ/Hsp40 family proteins, DNAJC5G, which actually assists the activity of Hsp70 by stabilizing their interaction with substrate proteins for their correct tertiary structure^34^, was also upregulated. These also strengthened our hypothesis that mahanine may have an immense effect on cellular protein folding machinery, especially affecting the ER which is predominantly assigned for this task.

ER is a key organelle to harbor intracellular Ca^2+^, in addition to other cellular perturbations that can generate ER stress. Decreased Ca^2+^ content in ER is an important stress factor inside this organelle^36^. Time-dependent changes in intracellular Ca^2+^ homeostasis and altered activity of Ca^2+^-dependent molecular pathways in treated cells also reinforce its functional assault in prime activities of ER^37^. It is already known that NFAT activates transcription of a large number of genes during an effective immune response^38^. Recently, it was proven that TNFα, which is known to induce apoptosis, also could be upregulated by NFAT-3^39^. Probably, mahanine-mediated NFAT-3 translocation from cytosol to nucleus helps in activation of apoptotic factors in PDAC cells.

Inhibition of Hsp90/Cdc37 induces IRE1 oligomerization, activation and hence induces ER stress^40^. This is also corroborated by our previous observation that mahanine treatment disrupts Hsp90/Cdc37 super-chaperone complex in PDAC cells^33^ and probably it would also assist to activate ER stress as we have observed in the current study. In this connection, it has been reported that Hsp inhibition is associated with activation of the UPR pathway in myeloma plasma cells^41^. Oxidative stress may induce ER stress in retinal pigment epithelial cells^42^ as we have observed in our study. Therefore it may be envisaged that mahanine-mediated ROS generation plays a critical role in ER stress activation in PDAC cells.

We revealed that mahanine could enhance the ROS-mediated activation of p53 family proteins in colon carcinoma cells^43^. Recent observation illustrated how ER stress stimulates p53 expression through NF-kB activation^44^. Our current finding adjoins a probable link between these two observations by addressing that mahanine-mediated ROS activated ER stress pathway and this would possibly strengthen the activation of p53 and its family proteins.

ER is the place where the glycosylation process starts. ER possesses several carbohydrate processing enzymes like UGGT, α-glucosidase-I/II, α-mannosidase along with lectin chaperones [CNX, CalR, and EDEM] for proper folding and degradation of glycoproteins. As we demonstrated that mahanine can mediate ER dysfunction, we explored the activity of this compound on perturbation of ER glycosylation mechanism.

Enhanced expression of sialic acid residues is directly related to the metastatic potential of human gastric cancer and neoplastic colon mucosa^27,28^. We also observed higher expression level of total α2–3-linked and α2–6-linked sialic acids on PDAC cells which were significantly downregulated after mahanine treatment. This was further corroborated by the reduced enzyme activity of sialyltransferases in treated PDAC cells while sialidases activity remained unaltered. In a very recent observation from our group, we also established that association of cytosolic Neu2 with membrane triggers Fas-mediated apoptosis by impairing PI3K-Akt/mTOR pathway in pancreatic cancer cells (in press).

Expression of gangliosides mainly depends on the status of sialic acid-modulatory enzymes, such as different types of sialyltransferases and sialidases. The increase of GD3 induces apoptosis through the mitochondrial pore formation or activating CD95/FAS^32,45^. We also demonstrated similar enhancement of GD3 in mahanine-treated PDAC cells suggesting another property of this prooxidant agent. This compound also induces apoptosis in different cancers by respective induction or inhibition of several pro- or anti-apoptotic pathways^43,46,47^. Another steroidal lactone (withanolide-D), from an Indian medicinal plant, showed attenuation of Wnt/β-Catenin pathway to restrain this PDAC disease^48^. Withanolide-D also target neutral sphingomyelinase-ceramide cascade in leukemia^49,50^. However, it’s role in these events remains to be investigated.

In this study, our data provide a molecular basis of employing ER stress in the apoptosis PDAC cell through a herbal alternative chemotherapy. In summary, mahanine causes apoptosis by enhancing ER stress through activation of several UPR pathways, calcium signaling and also attenuates the ER glycosylation along with overexpression of GD3 in PDAC cells. These results may endow a significant remedial strategy in the treatment of PDAC through ER stress-associated signaling pathways.

## Material and Methods

### Reagents

RPMI-1640, fetal bovine serum (FBS), antibiotic, antimycotic, bovine serum albumin (BSA), Ca^2+^ ionophore, EGTA, Thapsigargin (Tg), and Staurosporine (St) were purchased from Sigma Aldrich, USA. Anti-phospho-PERK (Thr^980^), PERK, phospho-eIF2α (S^51^), eIF2α, TRAF, IRE1α, BiP, Grp94, PDI, CHOP, Ero1-Lα, phospho-PKC βII (S^660^), phospho-PKC α/β (T^638^/^641^), Pan PKC, Calmodulin, NFAT-3, Calnexin, Caspase 12 and β-actin antibodies were purchased from Cell Signaling Technology, USA. NE-PER™ nuclear and cytoplasmic extraction reagents were from Pierce, Thermo-scientific, USA. ER-Tracker™ dyes for live-cell endoplasmic reticulum labeling was from Molecular Probes, USA. Biotinylated *Sambucus nigra* agglutinin (SNA), *Maackia amurensis*agglutinin (MAA), Peanut Agglutinin (PNA), Ricinus Communis Agglutinin (RCA) and Erythrina Cristagalli Lectin (ETC) from Vector LaboratoriesCA, US. Ca^2+^ Ionophore, EGTA, and N-acetyl-L-cysteine (NAC) were purchased From Sigma Aldrich

Mahanine was purified from fresh leaves of a native Indian plant, *Murraya koenigii* belonging to the family *Rutaceae*. The purity was confirmed by HPLC. LC-MS, [^1^H] and [^13^C] NMR spectral data analysis established its structure as mahanine^51^.

### Cell cultures

Human pancreatic adenocarcinoma (PDAC) cells MIAPaCa-2 and BxPC-3 were purchased from American Type Culture Collection, VA, USA and were grown in acomplete medium of RPMI-1640 (medium supplemented with 10% fetal bovine serum (FBS) and 1% antibiotic-antimycotic). Cells were cultured at 37 °C in an atmosphere of 5% CO_2_.

### Confocal microscopy

MIAPaCa-2 cells (5 × 10^3^) were seeded in 8 chambered polystyrene culture slide [BD Falcon, USA] per well in RPMI-1640 medium supplemented with FBS (10%). After 24 hrs of seeding, cells were exposed to mahanine (10–20 µM) along with vehicle control. Cells were washed with 1 × HBSS after 18 hr of incubation and processed for ER staining. Pre-warmed ER-Tracker Blue-White DPX (500 nM) was added to the cells and incubated for 30 mins at 37 °C at 5% CO_2_ incubator. The loading solution was removed and cells were then washed in HBSS. The samples were analyzed using confocal laser scanning microscope (NICON A1-R, NICON, Japan). Images were recorded using 60x/1.40 oil plan Apo-N objectives at calibrated magnification.

### Microarray analysis of using Illumina human Sentrix 6V2

A quantitative study was done using first strand cDNA by real-time PCR using a Light Cycler rapid thermal cycler system (Bio-Rad-Richmond, Richmond, CA) with SYBR Green Jump Start Ready mix (Sigma), following the manufacturer’s instruction. MIAPaCa-2 cell treated with mahanine (15 µM) for 18 hr. Total RNA was extracted using theRNeasy mini kit (Qiagen, Valencia, CA) and treated with a RNase free DNase I (Invitrogen) following the manufacturer’s instruction. First strand cDNA was synthesized by ImPromII-Reverse transcription system (Promega, Madison, WI). Isolated RNA was used for labeling, hybridization, and scanning of the Illumina human Sentrix 6V2 chip (San Diego, CA) in the Genomics and Proteomics Core Facility of the German Cancer Research Center according to Illumina’s recommended protocols. The Sentrix 6V2 bead chip includes an expression level of 48,600 human transcripts, variants, and EST clusters.

### *In vitro* intracellular Ca^2+^ measurement

MIAPaCa-2 cells (3 × 10^6^), treated with mahanine (20 µM), were washed in HBSS and then loaded with Fluo-3/AM (2.0 μM, Calbiochem, Germany) in HBSS containing CaCl_2_ (1.26 mM)^52^. The cells were incubated at 37 °C for 30 min in dark with gentle agitation. All extracellular Fluo-3/AM was removed by two-three times washing in the aforesaid buffer. The level of cytoplasmic Ca^2+^ within Fluo-3/AM loaded MIAPaCa-2 was determined in atime-dependent manner (0–2 hr) and analyzed with a FACS Calibur flow cytometer (Becton Dickinson, Mountain View, CA). The data were analyzed with the CellQuestPro software. (Becton Dickinson). The experiment was repeated in the absence of extracellular CaCl_2_. The mean fluorescence intensity (MFI) was measured. Ca^2+^ Ionophore (2 µM) and EGTA (10 mM) were used.

### Intracellular ROS measurement

Cells were treated with mahanine for 0–24 hr (20 µM) and 1hr (10–20 µM) and incubated with H_2_DCF-DA (10 µM) for 30 min at 37 °C. Intracellular H_2_O_2_ was determined using flow cytometry, by analyzing 10,000 cells with CellQuest Pro software (BD FACSCalibur). For ROS inhibition, the experiment was repeated with NAC (2.5 mM) pretreatment for 1 hr.

### Electrophoresis and Immunoblotting and immunoprecipitation

Human PDAC cells (1 × 10^6^) were incubated in complete medium alone, with standard dose of known ER stress inducer thapsigargin (1.0 µM) and staurosporine (1.0 µM) along with mahanine (10–20 µM) separately as indicated for 18 hr). Cells were detached using trypsin-EDTA solution. They were collected by centrifugation at 1500 g for 10 min and lysed by sonication. Aliquots containing total cellular proteins (60 µg) were separated by SDS-PAGE (10%) and transferred to nitrocellulose membrane (MILLIPORE, Bedford, MA, USA^53,54^). The membrane was blocked with TBS-BSA (2%-5%) for 1 hr at 25 °C and probed with desired primary antibody and β-actin (Cell signaling technology, USA) separately for overnight at 4 °C followed by HRP conjugated secondary antibody and detected by West-pico ECL system (Pierce, Thermo Scientific, USA). Additionally, the blots were also incubated with biotinylated-SNA (B-SNA 1:1000), biotinylated-MAA (B-MAA, 1:1000), followed by avidin-HRP (1:10000). The cells were treated with thapsigargin (1.0 µM) and staurosporine (1.0 µM) for 18 hr as the positive control for ER stress induction.

### Sub-cellular fractionation

PDAC cells (1 × 10^6^) were treated with mahanine (15 µM) for 18 hr and fractionated into cytosol and nuclear portions using an NE-PER® kit according to the manufacturer’s protocol. In brief, the treated cells were washed, incubated in cytosol extraction reagent, and centrifuged. The supernatant served as the cytosolic fraction. The pellet was solubilized in nuclear extraction reagent and centrifuged, and the supernatant represented the nuclear fraction. Western blot analyses were performed with these sub-cellular fractions as described previously.

### Flow cytometric analysis of cell-surface sialoglycoproteins

The expression of cell surface sialoglycoproteins was determined by flow cytometry^55,56^. Human PDAC cells (1 × 10^6^) were incubated in complete medium alone, with thapsigargin (1.0 µM) and staurosporine (1.0 µM) along with mahanine (10–20 µM) separately for 18 hr). Cells were harvested as described above and washed with phosphate buffer saline (0.02 M, pH 7.2, PBS). They were incubated with B-SNA, B-MAA, B-PNA, B-ETC and B-RCA) for 1 hr at 4 °C followed by probing with streptavidin-FITC antibodies. Cells were fixed in paraformaldehyde (1%) and analyzed with a flow cytometer and MFI was measured as described above.

### Sialyltransferases assay

The sialyltransferases activity in MIAPaCa-2 cell lysates was determined by a radiometric assay as described elsewhere^31,57^. Briefly, cell lysate protein (100 µg) was incubated with asialofetuin (6.0 nM) as an acceptor and CMP-[^14^C]Neu5Ac (0.5 µM) as a donor substrate, in cacodylate buffer (50 mM sodium cacodylate, 5 mM MnCl_2_, 150 mM NaCl, pH 6.5) with a total volume of 100 µL for 1 hr at 37 °C. The reaction was stopped by the addition of trichloroacetic acid (10%) and radioactivity was measured with a β-counter (Packard Bioscience Company, USA) using Cocktail-W (4.0 mL).

### Sialidase assay

The sialidase activity in MIAPaca-2 cell lysate was determined using a fluorimetric assay^30^. Briefly, cell lysate protein (100 µg) was incubated with 4-MU-Neu5Ac (30 nmol) as a substrate in a sodium acetate buffer (50 mM, pH 4.6) with a total volume of 100 µL for 1 hr at 37 °C. The reaction was stopped by the addition of a glycine/NaOH buffer (1.5 mL, pH 10.8). The fluorescence intensity was measured using excitation at 365 nm and emission at 450 nm.

### Semi-quantitative reverse transcription-PCR (RT-PCR) for GD3 expression

Total RNA was extracted from mahanine-treated (10–20 µM) PDAC cells using an RNeasy mini kit and reverse transcribed into cDNA with random primers using the Im-Pro-II-Reverse transcription system according to the manufacturer’s protocol. The GD3 and actin PCR assays were carried out with specific forward and reverse primers (Supplementary Table 1) using a PTC-100 system (MJ Research, MA, USA). The PCR products were electrophoresed on an agarose gel (1%), which was stained with ethidium bromide and visualized under UV light. The signal intensity of the respective DNA bands was measured with Quantity one version 4.1.1 software using a BIORAD image analysis system (CA, USA).

### Xenograft study ofa pancreatic tumor

Inbred female CD-1 nude mice of 4–6 weeks (20–25 gm of body weight) were housed in National Institute of Immunology (NII), India, fed a standard diet and were acclimated for 1 week in pathogen-free condition. All the animal-related experiments were performed in accordance with the National Regulatory Guidelines issued by Committee for the Purpose of Control And Supervision of Experiments on Animals (CPCSEA), Ministry of Environment and Forest, Govt. of India and used for experiments with prior approval from Institutional Animal Ethical Committee (IAEC) [IAEC approval no: IAEC # 257/11].

For subcutaneous xenograft study, mice were randomized into two groups; control and experimental, each group containing 5 mice. MIAPaCa-2 cells (1.5 × 10^6^) suspended in 100 μl of RPMI 1640-matrigel (BD Bioscience) in a ratio of 1:1, and injected subcutaneously into the flank of right hind limb and kept them for 25–30 days until nude mice developed tumors ranging from 100 to 150 mm^3^. Tumor growth recorded weekly in two dimensions using a Vernier caliper. Tumor volume calculated as [(length × width^2^)/2]. The mice were then kept either in vehicle [10% DMSO, 0.15 M NaCl injected as i.p] or in treatment [i.p. 100 mg/kg/day mahanine which is dissolved in 10% DMSO containing NaCl (0.15 M) solution] for minimum 17 successive days until the tumor load is significantly decreased. On the 18^th^ day, mice were sacrificed, tumor xenografts were excised from each mouse and required experiments were performed.

### Declaration for the experimental protocol

The authors confirm that all the *in vitro* works were very regular and routinely done in all labs. Relevant references have been cited in the Methodology section. All experimental methods were carried out in accordance with pertinent guidelines and regulations. This investigation is conforming to the Guide for the Care and Use of Laboratory Animals by the Committee for the Purpose of Control and Supervision of Experiments on Animals (CPCSEA) guidelines. All *in vivo* experiments were made in accordance with the relevant guidelines and regulations and were approved by Institutional Animal Ethical Committee (IAEC)of National Institute of Immunology (NII), New Delhi, India.

## Electronic supplementary material

Supplementary Information
