# Supplementary material for: Mahanine drives pancreatic adenocarcinoma cells into endoplasmic reticular stress-mediated apoptosis through modulating sialylation process and Ca2+-signaling
Source: Sci Rep. 2018 Mar 2;8:3911. doi: 10.1038/s41598-018-22143-w (PMC5834441; doi:10.1038/s41598-018-22143-w)
Supplement: Supplementary file 1 — Supplementary Information [file 41598_2018_22143_MOESM1_ESM.pdf]

Mahanine drives pancreatic adenocarcinoma cells into endoplasmic reticular stress-mediated apoptosis through modulating sialylation process and Ca^{2+}-signaling

Sayantani Sarkar Bhattacharya[1], Chandan Mandal[1], Reinhard Schwartz Albiez[2], Suman Kumar Samanta[1] and Chitra Mandal[1*]

[1]Cancer Biology and Inflammatory Disorder Division, Council of Scientific and Industrial Research-Indian Institute of Chemical Biology, Kolkata 700032, India

[2]German Cancer Research Center Heidelberg, Antigen Presentation & T/NK Cell Activation, Clinical Cooperation Unit Applied Tumor Immunity, ImNeuenheimer Feld 280, D-69120 Heidelberg, Germany

*** Corresponding Author**

Prof. Chitra Mandal

Cancer Biology and Inflammatory Disorder Division

CSIR-Indian Institute of Chemical Biology,

4, Raja S.C. Mallick Road, Kolkata 700032, India

Mobile: 09831036964; Telephone: 033-2429-8861

E mail: chitra_mandal@yahoo.com; cmandal@iicb.res.in

Fig S1

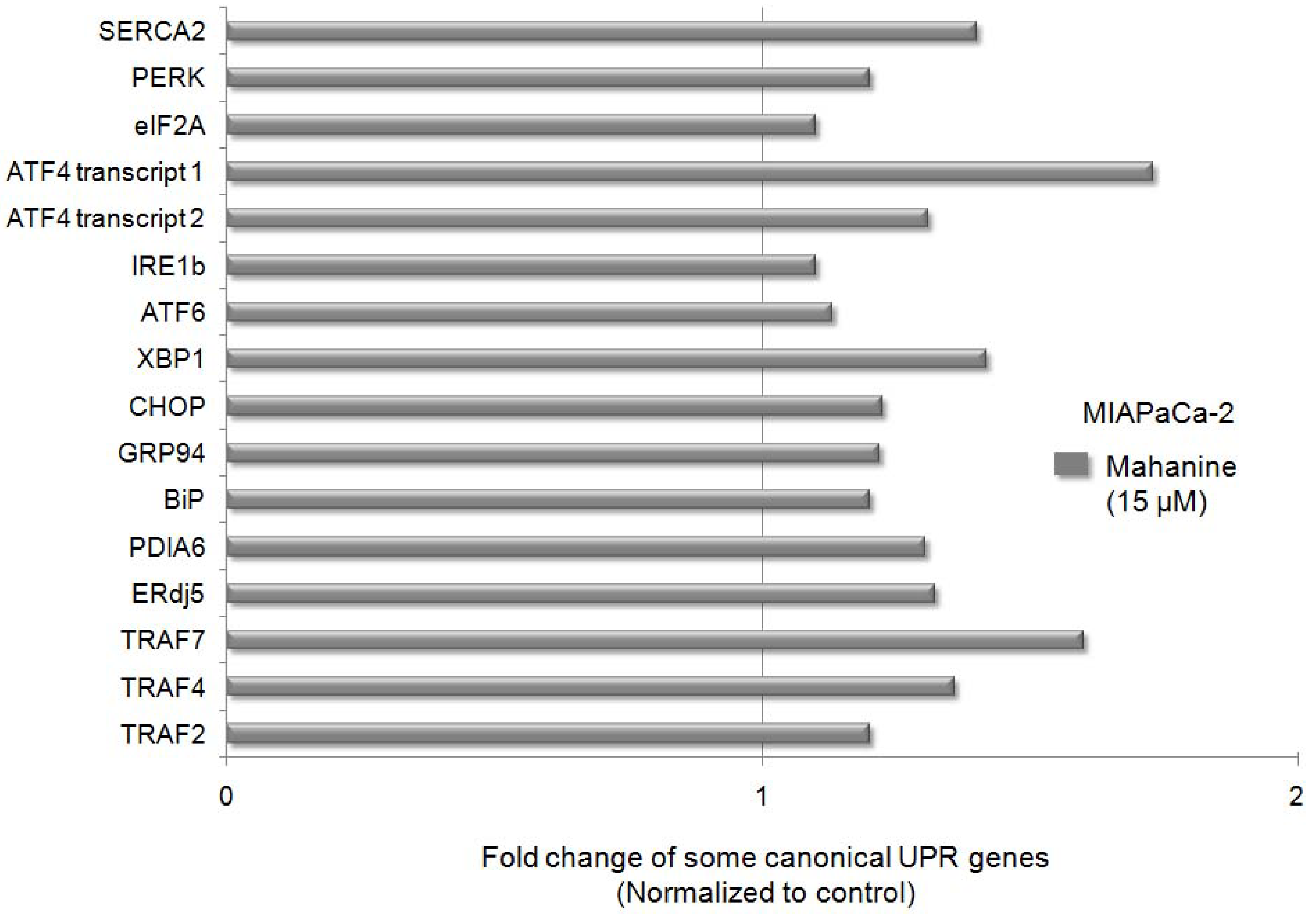

Figure S1. Mahanine initiates unfolded protein response in pancreatic carcinoma cells. Fold changes in most of the UPR genes in mahanine-treated (15 µM, 18 h) MIAPaCa-2 cells demonstrated in microarray analysis as describe in material methods. One-way ANOVA was used to analyze the data.

Fig S2

A

Gene Symbol	Fold change
DNAJC5G	1.86
DNAJB6	1.76
DNAJC6	1.46
DNAJC7	1.44
DNAJC11	1.44
DNAJB14	1.39
DNAJB2	1.33
DNAJB14	1.28
DNAJC30	1.27
DNAJB14	1.23
DNAJB12	1.22
DNAJA1	1.21
DNAJA4	1.15

B

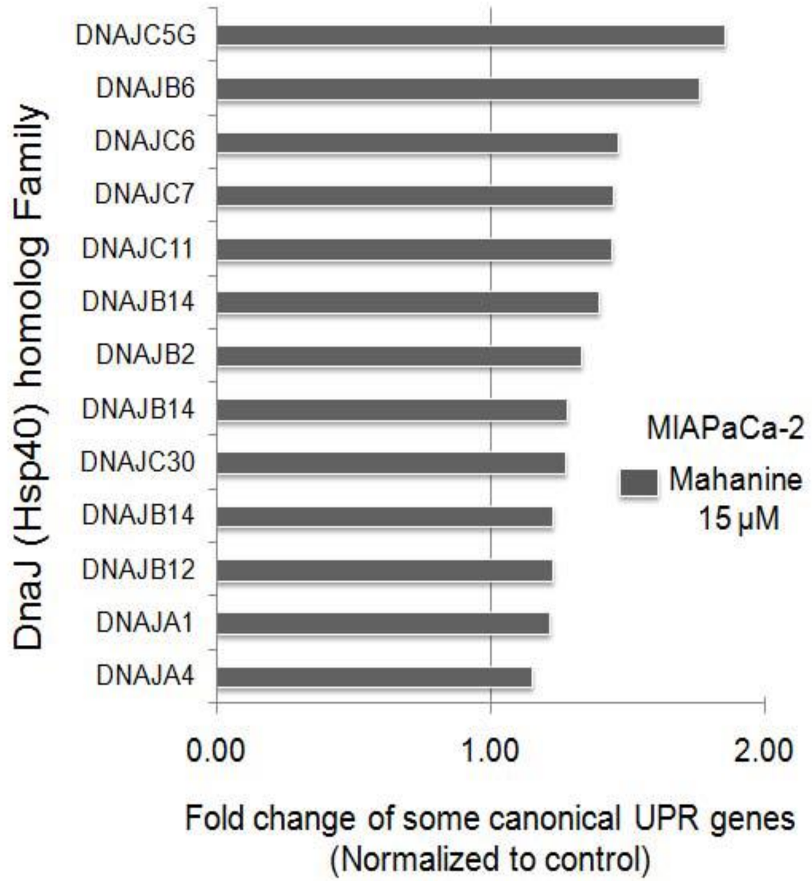

Figure S2. Mahanine mediated augmentation of DnaJ or Hsp40 super family gene transcripts in MIAPaCa-2 cells. Tabular **(A)** and graphical **(B)** representation of few significantly changed DnaJ transcripts from the microarray data analysis in mahanine-treated (15 µM, 18 hr) MIAPaCa2 cells in compare to control as describe in material methods.

Fig S3

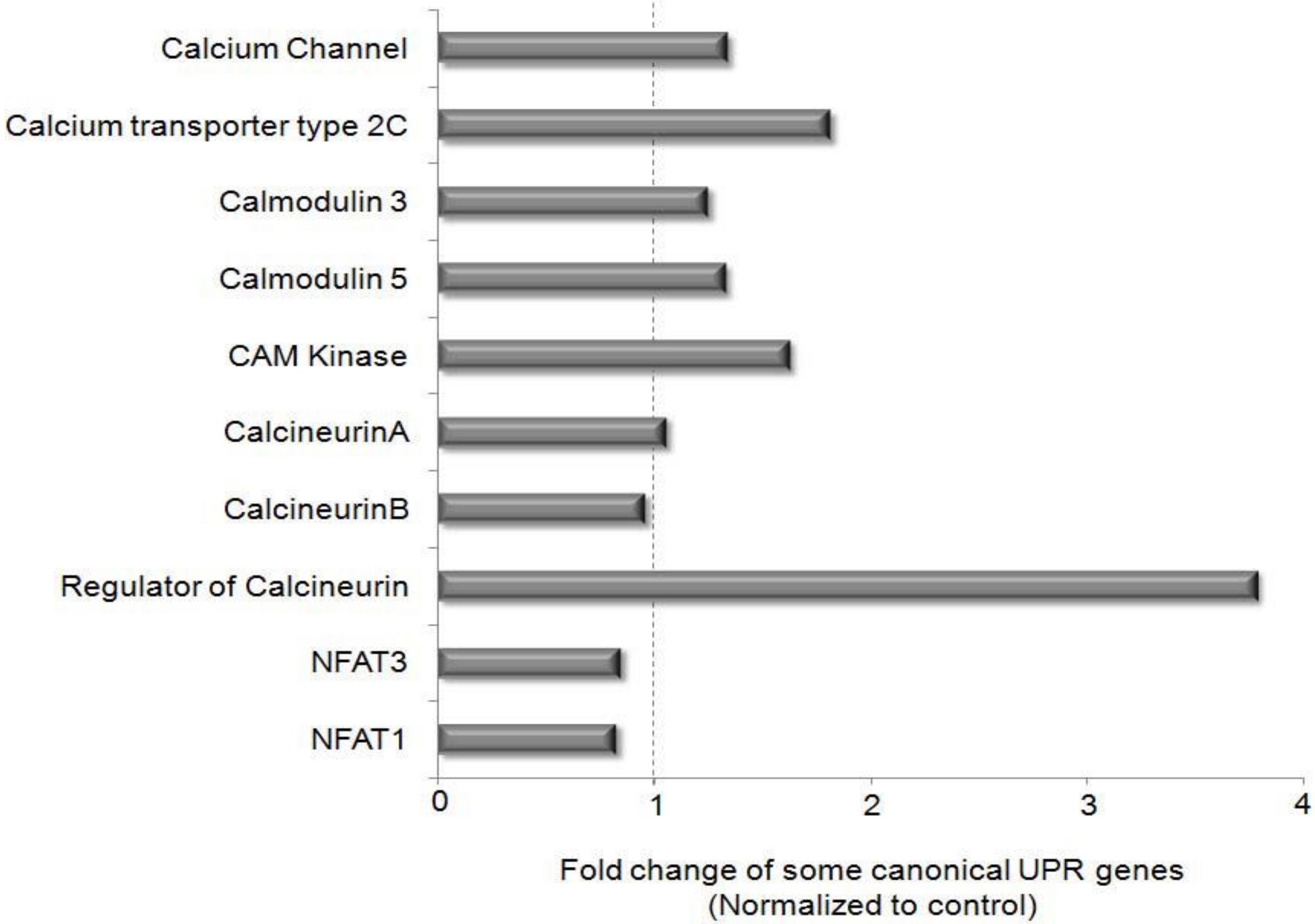

Figure S3. Mahanine induced upregulation of gene transcripts involved in Ca^{2+} signalling. Data represented as fold change in genes expression from the microarray analysis in mahanine-treated (15 µM, 18 hr) MIAPaCa2 cells in comparison to control.

Fig S4

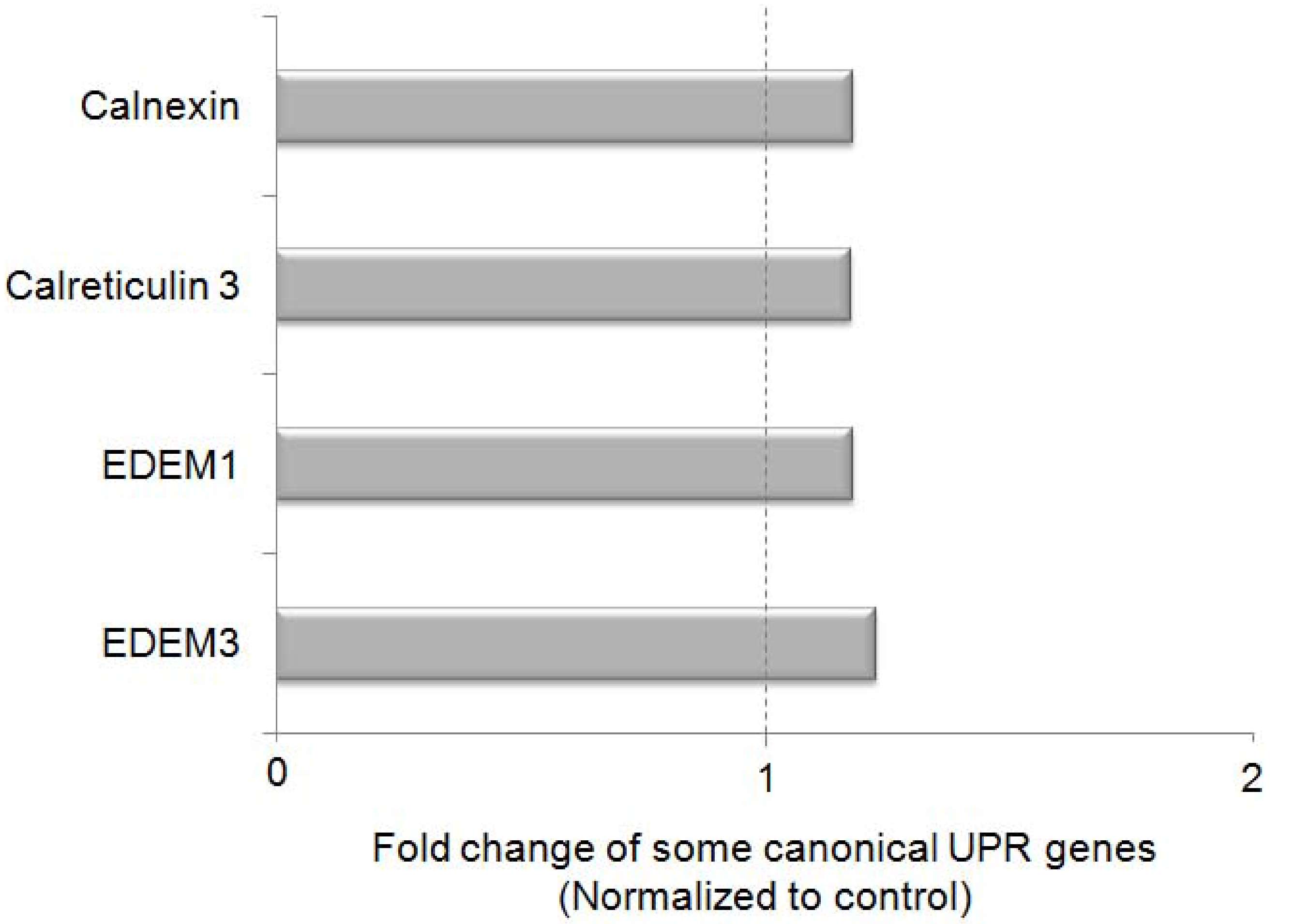

Figure S4. Microarray data of gene transcripts involved in Calnexin-Calreticulin chaperone cycle. Data represented as fold change in genes expression from the microarray analysis in mahanine-treated (15 µM, 18 hr) MIAPaCa2 cells in comparison to control.

Fig S5

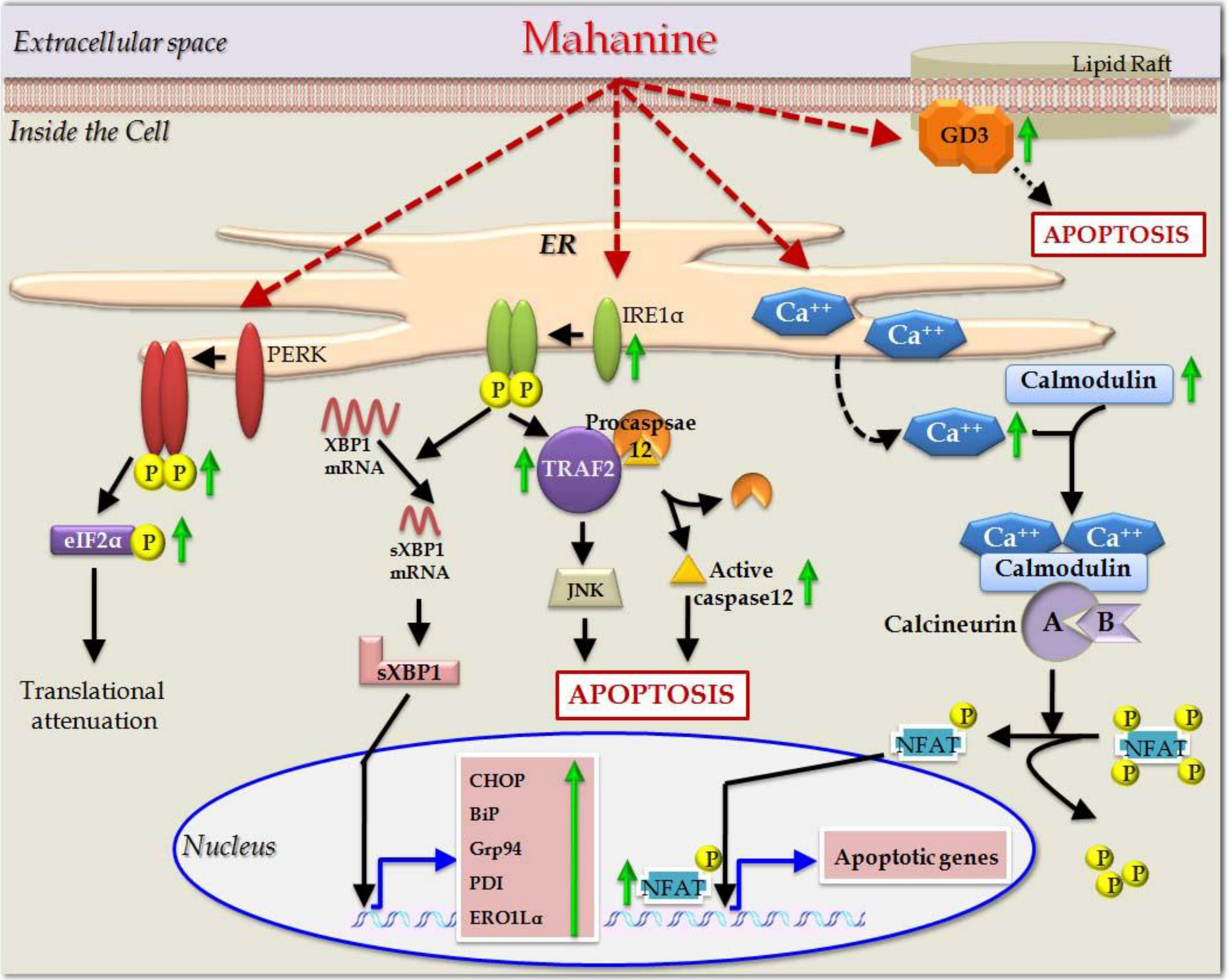

Figure S5. Schematic diagram of the summarized activity of mahanine on UPR activation and ER stress.

Table S1

Gene Symbol	Fold change
SEC23A	2.1
ERP29	1.55
ST8SIA1	1.52
CREB3L4	1.44
CREB3L1	1.4
CREB3L2	1.35
SELS	1.35
COPA	1.35
HERPUD1	1.34
FKBP7	1.34
ERAP2	1.34
CREB3	1.32
PDIA6	1.31
ST8SIA1	1.27
HSP90B1	1.23
EDEM3	1.23
CLGN	1.22

Table 1: Changes in expression levels of a few genes in the classic UPR pathway
